# Deaf nursing professionals: experiences of social interaction and communication in professional practice^*^

**DOI:** 10.1590/1518-8345.8211.4912

**Published:** 2026-08-13

**Authors:** Aline de Barros Campanelli Lima, Bárbara Barrionuevo Bonini

**Affiliations:** 1 Instituto Israelita de Ensino e Pesquisa Albert Einstein, Unidade Paulista II, São Paulo, SP, Brazil.

**Keywords:** Deafness, Nursing, Social Inclusion, Professional Practice, Social Interaction, Communication.

## Abstract

**Objective:**

to explore the inclusion of deaf nursing professionals in terms of attitudinal and communicational accessibility in the workplace.

**Method:**

qualitative, cross-sectional, descriptive study based on the history of the inclusion of people with disabilities and Sassaki’s seven dimensions of accessibility. Semi-structured online interviews were conducted with deaf nursing professionals. The analysis followed Braun and Clarke’s Thematic Analysis method.

**Results:**

eight professionals participated, including six nurses and two nursing technicians, with mild-to-profound hearing loss. Two main themes emerged-Accessibility and Barriers-and four subthemes: attitudinal accessibility (representation, support, and inclusive coexistence) and communicational accessibility (use of adapted communication strategies); attitudinal barriers (ableism, invisibility of deafness, and discrimination) and communicational barriers (protective masks, noisy environments, use of writing, and auditory fatigue). Despite occasional advances, structural and cultural barriers persist that hinder the full inclusion of these professionals.

**Conclusion:**

the inclusion of people with hearing loss in nursing remains partial, particularly in attitudes and communication. The presence of these professionals contributes to raising awareness about deafness, reducing stigma, and strengthening accessibility practices, all of which are essential to a more diverse and equitable nursing profession.

## Highlights

**(1)** The inclusion of people with hearing loss in nursing is still partial. **(2)** The presence of deaf professionals contributes to raising awareness about deafness. **(3)** This presence helps reduce inequalities in access to healthcare. **(4)** With the necessary adaptations, communication becomes feasible. **(5)** Collaboration between society and deaf nursing professionals is essential.

## Introduction

According to the 2022 Continuous National Household Sample Survey (*PNAD Contínua*, acronym in Portuguese), 1.2% of the country’s population, or 2.51 million Brazilians, have difficulty hearing even with a hearing aid^1^. However, this segment of the population still finds it difficult to enter various social spaces, such as the job market^1^. PNAD also reports that among individuals with hearing difficulties, 24.4% are employed. According to the 2023 Annual Social Information Report (RAIS, acronym in Portuguese), the number of people with hearing loss (HL) in the formal labor market was 113,471, representing 0.21% of the country’s total workforce^1^.

This scenario of low inclusion is repeated in the health sector and among those who wish to enter nursing^2^. In addition, there are no statistical data available on the total number of deaf nursing professionals in the surveys conducted by professional associations. Finally, according to the Higher Education Census, between 2010 and 2023, among students who graduated in nursing, only 0.04% had HL and 0.01% were deaf^3^.

As such, little is known about the lived realities of these professionals, particularly what it is like to work in nursing while living with HL. In view of this, we sought to explore the inclusion of deaf nursing professionals with respect to attitudinal and communication accessibility in the workplace.

## Method

### Study design 

This is a descriptive, cross-sectional study with a qualitative approach^4^.

### Theoretical framework

Social Inclusion is a movement in favor of people with disabilities (PWD) that began in the late 20th and early 21st centuries. Its goal is to establish a society that is truly for everyone, based on six principles: the celebration of differences, the right to belong, appreciation of human diversity, humanitarian solidarity, the equal importance of minorities, and citizenship and quality of life. This requires cooperation between society and PWD^5^.

However, social practices regarding PWD have undergone various phases over the years. Initially, various cultures practiced social exclusion of individuals with atypical conditions, separating them, institutionalizing them, or even eliminating them, since they were considered invalid. Then, specialized entities began to emerge for each type of disability, such as special schools, habilitation and rehabilitation centers, sheltered workshops, among others, giving rise to Institutional Segregation. Beginning in 1960, Social Integration emerged, which did not aim at the full participation of individuals with disabilities in social sectors, but rather at their insertion into social spaces without adequate adaptations. PWD who began to actively participate in society did so through personal merit, thereby achieving defined social standards^5^.

For Sassaki, there are seven dimensions of accessibility, designed to clarify the practical application of the concepts proposed in the inclusion movement and to help identify and mitigate accessibility barriers^6^. These are: architectural, attitudinal, communicational, instrumental, methodological, natural, and programmatic. In this investigation, the attitudinal and communicational dimensions in workspaces are highlighted. The first deals with access without barriers resulting from prejudices, stigmas, stereotypes, and discrimination that prevent or hinder the social participation of people with disabilities on an equal basis with others^6^ ^-^ ^7^. The second concerns barrier-free access to communication in its many forms: written, verbal, gestural, Brazilian Sign Language (*Libras*, in Portuguese), facial, body language, in person or remotely, in healthcare, and others^5^.

### Location and period of data collection

Data were collected via Zoom interviews from August 15 to December 11, 2024. During the interviews, both the participants and the interviewer were at home, in a private room.

### Participants

The participants were deaf individuals aged ≥ 18 years, nursing professionals (nurses, technicians, or assistants) who were actively registered with a professional association and working in any area of nursing. The sampling technique used was snowball sampling^4^.

### Data collection instrument

We decided to conduct semi-structured interviews via Zoom Meeting, led by the first author, a student in the professional master’s program in nursing, with guidance from the second author, her advisor. A pilot interview was conducted because this was the student’s first experience, to test and inform possible adjustments.

The average interview duration was 40 minutes, including the interviewer’s introduction (name and institution), explanation of the objectives, clarification of questions, and closing remarks. The conversations were recorded on video and audio using the platform’s own resources. Notes were also taken during the interviews, such as the interviewer’s impressions.

The interview script was divided into two parts: demographic characterization and guiding questions (Figure 1).

**Figure 1:** Interview script

Questions	Alternatives	
What is your professional category?	Nurse	Nursing technician
	Nursing assistant	
What is your degree of hearing loss?	Mild	Moderate
	Severe	Deep
When did your hearing loss begin?	Pre-lingual	Post-lingual
Are you oralized?	Yes	No
What is your first language?	Portuguese	Sign language
Do you know sign language?	Yes	No
Do you use a hearing aid?	Yes	No
Do you read lips?	Yes	No
Age:		
City where you work:		
Year of technical or undergraduate degree:		
Length of time working in nursing:		
What level of healthcare do you work in?	Primary level	Secondary level
	Tertiary level	
Your work is part of the initiative:	Public	Private
What was it like for you to enter the job market as a deaf person?		
Were any accommodations made to welcome you where you worked/work?		
Do you think there are any advantages to being a deaf nursing professional? If so, what are they?		
Do you think there are any challenges to working as a deaf nursing professional? If so, what are they?		

The interviews were conducted only once with each participant at a date and time of their choice. Only one participant, who communicated exclusively through Libras, required an interpreter.

### Data collection 

The entire database was stored on the Research Electronic Data Capture (REDCap) platform.

The initial intermediaries, or “seeds”, were individuals with whom the researcher already had prior contact and who could even become participants^4^. To invite other participants, the seeds received a specific message about the project and a link to complete the Free and Informed Consent Form (FICF). After accepting, the participant provided three days and times for the interview and indicated whether a sign language Libras interpreter was needed during the conversation.

Those who agreed to participate in the study were then asked to invite other people^4^. Data collection was considered complete when no additional participants were recruited, and it was understood that the data obtained already met the proposed objective.

Seventeen REDCap accesses were recorded, but only eight resulted in valid interviews. Eight attempts were incomplete at the FICF step, and one participant was excluded for lacking a nursing degree, having been advised on the inclusion criteria.

### Data analysis

First, the interview recordings were uploaded to Transkriptor, a software tool that uses Artificial Intelligence (AI) to transcribe audio into text^8^. However, to ensure data quality, all materials were reviewed multiple times to correct errors. The transcripts were not returned to the participants.

In accordance with the principles of Open Science, all datasets in this article, i.e., the full interviews, are available in the SciELO Data repository under the same name as this article, via the link https://doi.org/10.48331/scielodata.0FSQFM.

Data analysis was conducted using Braun and Clarke’s thematic analysis. This process was carried out using the Taguette platform, a free tool that facilitates coding, categorization, pattern identification, and reports generation^9^. 

Familiarization with the database occurred during the transcription of the interviews, followed by a complete reading of the material. Phases two and three involved the creation of initial codes and themes, with a focus on the individual characteristics of each interview. In phase four, a new analysis was performed to refine the categories, considering the set of interviews. The relationship between the categories and the seven dimensions of accessibility proposed by Sassaki was then examined, leading to a new analysis to assess the adequacy of the findings for these dimensions^10^.

### Ethical considerations

The study was conducted in accordance to national and international ethical guidelines and was approved by the Research Ethics Committee of Hospital Israelita Albert Einstein, CAAE: 77221424.0.0000.0071, opinion no. 6,885,669.

Participants were identified by an alphanumeric code (e.g., P1, P2...) and sensitive information was omitted from the transcripts.

## Results

In the end, eight interviews were conducted. The ages ranged from 26 to 39 years, with an average age of 31 years. Six participants were nurses, and two were nursing technicians.

The degrees of HL reported ranged from mild to profound, with only three acquiring the condition before learning to speak. Only one participant had *Libras* as their first language rather than Portuguese. This same individual was the only one who communicated solely through Libras. 

Two participants reported knowing *Libras*, three reported knowing only the basics, and three had no knowledge of this language. Only two individuals did not use hearing aids; the other five used them in both ears. All interviewees reported lip-reading, using this strategy infrequently or frequently, depending on their needs.

Regarding participants’ nursing careers, the length of service ranged from eight months to 17 years, with a mean of 6.8 years. Four worked in the tertiary health care sector, and one worked in the secondary sector. For three participants, this question did not apply, as one worked in the government health sector and two were teachers. Finally, the participants were from the Northeast, South, and Southeast regions of Brazil.

In total, the analysis underwent three iterations until a satisfactory result was achieved. Of the seven dimensions of accessibility. However, this article focuses solely on the attitudinal and communicational dimensions. These dimensions were then divided into accessibility and barriers. It is essential to emphasize that the themes were constructed based on the results obtained and were not previously established. In addition, the final descriptions of these were based on the concepts presented by Sassaki and adapted according to the needs of the analysis.

The themes are Accessibility and Barriers. The first concerns reports in which contexts aligned with inclusion efforts were identified. The second concerns reports in which barriers to inclusion were identified. 

The first theme gives rise to the sub-themes Attitudinal Accessibility, with 34 codes, and Communication Accessibility, with 40 codes. The theme Barriers gives rise to the sub-themes Attitudinal Barriers, with 19 codes, and Communication Barriers, with 24 codes.

### Attitudinal accessibility

Demonstrates attitudes and behaviors that promote inclusion, such as representation, support, and equal coexistence. Some examples of codes are:

*In the end, I said, “[...] I am his nurse, but I am also deaf.” [...]. That child’s grandparents arrived and said, “HL will not prevent my grandson from being who he wants to be.” (P6)*

*[...] I think that when a person wants something and has other people who help and adapt, it is always possible. (P5)*

*Often, the girls [nursing team] told me that it was only after they had contact with me that they knew how they would act with these patients [deaf patients], with the families of these patients. (P6)*

*[...] today, all my patients, when I say, “I am deaf [...],” they have all accepted it. (P6)*

*I believe that I would not have the level of empathy that I have today if I had not had this condition. (P1)*

### Communication accessibility

Demonstrates the establishment of communication between the participant and other professionals, patients, and their families through the adoption of different strategies. Some examples of codes are:

*[...] when I arrived in the sector, I had to tell everyone [...] “I don’t hear well, if you want to talk to me, talk to me face to face or touch me, then I can talk to you, because if you talk to me from behind, I won’t be able to understand.” (P1)*

*[...] I said, “Look, since you think my deafness is having such an impact, from now on, I request that you give me all patient information in writing.” (P5)*

*I went to see a patient and asked if he could take off his mask, keep only the visor [transparent face shield], and we would keep our distance. He was afraid, but he took it off. (P2)*

*When I communicate with elderly people, I use more gestures, because they are already old and can’t read because of their vision. (P7)*

### Attitudinal barriers

They demonstrate attitudes and behaviors that are unfavorable to inclusion, such as ableism, invisibility of deafness, and discrimination. Some examples of codes are:

*And in the workplace too, because unfortunately there is ableism where people think that because you are a person with a disability, you are incapable or less capable. [...] You don’t have as much ability as someone who can hear. (P1)*

*I think they need to be more inclusive when they welcome us, and not look down on us, because even though it may not seem like it, I am still disabled. (P3)*

*I heard [...] that some colleagues asked if I could go to another department because I wouldn’t be able to work in that department since I was a person who couldn’t hear. (P1)*

*So I had some difficulties, really, with these issues [understanding of other health professionals regarding the HL], and I asked for help from the coordination team. Was you well attended to? Not necessarily. (P3)*

*[...] there was a patient who wanted to have her blood pressure checked [...] and she said, “Wow, there’s only one nurse in the clinic and they put a deaf one on me?” (P5)*

*To advocate about this is tiring, very tiring. (P2)*

### Communication barriers

These demonstrate communication barriers caused or aggravated by hearing disabilities, such as the use of protective masks, noisy environments, the use of writing, and auditory fatigue. Some examples of codes are:

*[...] it was a [hospital] environment where everyone was wearing masks, so my ability to read lips was useless there. (P3)*

*A hearing aid will never be an ear. The device greatly amplifies all sounds, all of them! So the noise from the air conditioner, for me, will be very loud, but the noise from voices, for me, will not be loud. (P3)*

*[...] I see that there is still a certain barrier. For example, I work in hemodynamics, and we know that [...] there are noises. There are pump alarms, monitor alarms, cardioverter alarms [...]. (P6)*

*Besides, you know that deaf people often write Portuguese like foreigners. It’s not the conventional Portuguese that everyone else writes. (P7)*

*[...] a person who does not hear well and is a healthcare professional is, unfortunately, already a much more anxious and tired person. [...] because we make a greater effort than other people to understand information [...]. (P4).*

*My biggest challenge is working in the most chaotic sectors, such as the ICU and emergency room, areas that are very busy, where people often don’t have time to stop and talk to me, to pay attention. (P7)*

## Discussion

The discussion of the topics was organized by dimension. Initially, the forms of accessibility observed will be presented, followed by the barriers associated with each. This structure allows a direct comparison of the two scenarios, providing a clearer, more integrated view of each analyzed dimension.

### Attitudinal accessibility

A frequent theme in the reports was scenarios of representativeness. Also known as identity representation, this refers to people who serve as references for a population, since they share characteristics with the individuals represented^11^. The presence of these individuals in social spheres increases society’s awareness of PWD and inclusion, as indicated in P6’s interaction with a child’s family.

Sometimes, patients are afflicted by conditions that may be experienced by the very professionals who provide their care. In other words, inclusion and diversity in healthcare bring substantial gains in patient care and experience^12^. It is also important to note that the presence of professionals whose language and culture are the same as those of their patients helps to reduce inequalities in access to healthcare. Additionally, it aids in this process by promoting education and awareness among other professionals about PWD^13^.

Another point that was consistently emphasized was the importance of receiving support from other professionals and institutional coordinators. This support, especially when given genuinely and not because the individual is considered incapable or pitying, promotes greater self-confidence and stability in professionals with disabilities^14^. Sassaki states that one of the consequences of inclusion is that this process ultimately benefits not only individuals with disabilities, but also the community^5^ ^-^ ^6^, as exemplified by participants 5 and 6.

As regards patients’ acceptance of care provided by a healthcare professional with HL, although there are reports of negative responses, most interviewees reported positive experiences, as exemplified by P6. In a study involving deaf healthcare professionals with from different areas, it was found that patients generally showed neutral or positive reactions to the care provided by these professionals^12^.

In addition, one characteristic of the care provided is professionals’ empathy toward their patients. A study found that medical students with various disabilities were less likely to have low levels of empathy^15^, a characteristic P1 identified as a positive aspect of deafness that informed his nursing practice. Thus, the findings of both studies are consistent with the results obtained in this investigation.

### Attitudinal barriers

Anyone can face challenges in the job market. However, people with disabilities experience minority tax. This means that, in addition to the natural responsibilities and difficulties of this environment, people with disabilities face additional barriers due to prejudice, isolation, and exclusion, among others^12^.

One component of this phenomenon is ableism. This term refers to judging and discriminating against a person based on their physical and mental abilities and using this to classify them as incapable^2^. Unfortunately, some interviewees, such as P1, reported having experienced this phenomenon. It is important to emphasize that this is a structural construction of society, the result of a historical system of policies, institutions, and social norms that devalue and marginalize people with disabilities^2^. Reports indicate that it can stem from institutional initiatives, such as limited access to communication, or from the individual level, through negative attitudes, including the use of discriminatory language.

Studies on discrimination and ableism suffered by healthcare professionals with disabilities prove that this scenario is recurrent. In addition, it can discourage these individuals from working in the healthcare system or even impact their well-being^2^. Fortunately, among the participants in this study, although it caused some annoyance, there were no major complications attributable to ableist attitudes.

Another relevant factor is the invisibility of HL. It is impossible to distinguish a deaf individual from others unless they choose to disclose this information. Occasionally, the use of hearing aids is one of the few ways to make this characteristic visible^16^. One of the interviewees (P3) reported that, due to the invisibility of deafness, others mistakenly perceived that HL has minimal impact on their professional lives. 

This report indicates that the invisibility of deafness can lead to subtle forms of denial of disability, reinforcing ableism and creating additional obstacles to the full inclusion of deaf people in society.

Another obstacle, part of the minority tax, and one of the most important, is discrimination. Several reports of discrimination were made in interactions with other health professionals (P1), institutional coordination (P3), patients and their families (P5).

These reports reflect the lack of PWD in social spaces and people’s ignorance about these individuals. There are several stereotypes about the abilities of deaf people, such as being less intelligent, having fewer communication skills, lacking social skills, and being unable to write, among others^12^. Sassaki proposes that one of the main ways to address discrimination is through education about diversity and the inclusion of people with disabilities across all spheres of society. One of the recommendations is to implement awareness programs for employees, which have shown positive effects in reducing attitudinal barriers in the workplace^6^.

Finally, it is essential to emphasize that this awareness needs to be raised through organizational initiatives, since it is often left to individuals with disabilities to provide this education in loco and to advocate for themselves^6^ ^,^ ^12^. The need for self-advocacy is one of the components of the minority tax and, as it is a constant demand, it becomes exhausting^12^, as mentioned by P2. In this sense, it is worth remembering that true inclusion requires collaboration and mutual effort between PWD and society^5^.

### Communication accessibility

An important theme identified was the incorporation of communication strategies tailored to each participant’s specific needs, as exemplified by P1. It is possible to identify that, in certain situations, small, easily implemented actions, such as standing face-to-face when speaking with a deaf person, can make communication more accessible. However, it should be noted that, once again, it was observed that PWD are responsible for raising awareness and educating others about the methods available to promote inclusion, as many lack this knowledge^5^ ^,^ ^12^. This reinforces the asymmetry in the effort made to achieve inclusion, as described by Sassaki in his analyses of integrative practices^5^ ^-^ ^6^.

A specific feature of nursing professionals’ routines is the shift handover, which is typically conducted verbally. However, an adjustment was made to the information transmission process after interviewee P5 reported difficulties with it, given that the scenario involved multiple people speaking simultaneously.

This example reaffirms findings from a study of individuals with unilateral HL, which emphasized that noisy environments impair speech comprehension^17^ ^-^ ^18^. Furthermore, in a scenario such as the one described by the participant, with multiple people speaking at once, it is not possible to lip-read everyone. Therefore, changes such as the one mentioned above can enhance security for both the professional and the patient, as effective communication is crucial for adequate care^19^.

Finally, the interviewees reported some strategies they developed to establish effective communication with their patients, such as lip reading and gestures in the cases of P2 and P7, respectively. Studies present other strategies used by deaf healthcare professionals to establish communication with their patients. Examples included: microphone systems, inclusive masks, even if not standardized, interpreters, explanatory cards, and simultaneous text transcription applications^19^.

It can be inferred that the choice of strategy depends on multiple factors, including resource availability, the professional’s needs, and each patient’s specific characteristics. Thus, there is no way to establish a standardized ideal method. Therefore, various forms of communication should be available so that the professional can choose those that best apply to each case.

### Communication barriers

Various nursing environments involve the use of protective masks most of the time. During the COVID-19 pandemic, this use became even more necessary and occurred in situations that were not common in daily life, resulting in significant impacts on people with hearing loss^20^.

As exemplified by P6, wearing a mask impacts an individual’s ability to read lips, which is often a complement to communication. For deaf people who use Libras, wearing a mask impairs the perception of facial expressions, which are important in sign language^20^. Thus, the use of masks generated higher levels of stress and anxiety in healthcare deaf professionals. Some participants emphasized that this made them fearful about their future careers, prompting them to question whether they would need to change professions, as the barrier seemed impossible to overcome. Therefore, this barrier also undermined these professionals’ sense of autonomy, independence, and belonging, fostering feelings of isolation and an inability to demonstrate professional competence^20^.

Another factor that impairs communication is auditory perception in noisy environments. According to participants, examples include operating rooms, emergency rooms, ICUs, and cardiac catheterization laboratories. Individuals who use hearing aids often report difficulty with auditory speech discrimination in acoustically challenging environments. In these instances, the individual must expend greater cognitive effort to discern speech from other noises. This effort requires a high level of concentration, which is then unavailable for other activities^21^.

Regarding the use of writing as a communication strategy, it is important to note that although it can be helpful in some situations, this method may not be entirely effective. Individuals with deafness who have written Portuguese as a second language may have difficulties with this mode of communication due to the semantic and grammatical differences between Libras and Portuguese^19^. In the case of individuals with HL who have Portuguese as their first language, it is assumed that they may have difficulties learning the language, both spoken and written, because of HL^17^ ^-^ ^18^. In light of this, written communication should not be considered the primary means of establishing effective communication with a deaf individual^19^.

Given these communication barriers, participants such as P4 reported experiencing mental fatigue from the sustained effort required to communicate. Auditory fatigue is also reported by individuals with deafness after exposure to noisy environments, that require interactivity and, therefore, greater cognitive and concentration load. As a result, it can lead to higher levels of stress^17^ ^-^ ^18^. However, this mental fatigue was not the only consequence identified in communication barriers. Participants, such as P7, reported that certain areas of nursing practice were now considered infeasible for professionals with HL due to barriers encountered, particularly those related to communication.

 This account exemplifies the integration phase, since deaf people are entering environments without receiving the necessary accommodations. Thus, these individuals need to adapt as best they can^4^. It is common for institutions to inadvertently discourage these individuals from pursuing subspecialty training. This is due to both the failure to provide necessary accommodation and the belief that these individuals should not, or would not, be able to work in settings where they are in contact with patients or where communication must be rapid. However, with adequate resources, people with HL can perform their profession with quality and safety^12^.

This study was limited by a lower-than-expected number of participants, with an estimated 15 participants. However, this difficulty was anticipated given the limited access to this population^22^. In any case, it is believed that it was possible to obtain significant results on the subject and bring an innovative perspective on the performance of deaf nursing professionals, since any proposal made about PWD must involve them.

## Conclusion

When comparing Sassaki’s concept of inclusion with participants’ experiences regarding attitudinal and communicative accessibility, full inclusion of deaf people in nursing remains far from realized. Society’s ignorance about PWD and the means available to mitigate barriers are among the main causes of the challenges identified.

However, positive reports show that this is a reality in the making. To this end, it is worth emphasizing that it is imperative for society to take a more active role in the process of including people with disabilities, so that the burden does not fall solely on them.

On the other hand, the presence of people with hearing disabilities in nursing contributes to raising society’s awareness about PWD, showing that disability does not prevent people from working in nursing, breaking down stigmas and prejudices, and reinforcing the importance of implementing accessibility strategies. Consequently, this presence helps to reduce inequalities in access to health care for other people with disabilities. In terms of communication, it is possible to understand that, with the necessary adaptations, communication becomes feasible for professionals to perform their duties safely, autonomously, and with less mental effort.

## Data Availability

The dataset of this article is available on the RLAE page in the SciELO Data repository, at the link https://doi.org/10.48331/scielodata.0FSQFM.
