# Supplementary material for: Deaf nursing professionals: experiences of social interaction and communication in professional practice
Source: Rev Lat Am Enfermagem. 2026 Aug 13;34:e4912. doi: 10.1590/1518-8345.8211.4912 (PMC13505606; doi:10.1590/1518-8345.8211.4912)
Supplement: Translation

# Translation: Profissionais da enfermagem surdos: experiências de interação social e comunicação na prática profissional^*^

**DOI:** 10.1590/1518-8345.8211.4913

**Authors:** Aline de Barros Campanelli Lima, Bárbara Barrionuevo Bonini

**Affiliations:** 1 Instituto Israelita de Ensino e Pesquisa Albert Einstein, Unidade Paulista II, São Paulo, SP, Brasil.

**Keywords:** Surdez, Enfermagem, Inclusão Social, Prática Profissional, Interação Social, Comunicação

## Abstract

**Objetivo:**

explorar a inclusão de profissionais da enfermagem surdos nas dimensões da acessibilidade atitudinal e comunicacional no ambiente de trabalho.

**Método:**

estudo qualitativo, transversal e descritivo, fundamentado na história da inclusão de pessoas com deficiência e nas sete dimensões da acessibilidade de Sassaki. Foram realizadas entrevistas semiestruturadas *online* com profissionais da enfermagem surdos. A análise seguiu o método de Análise Temática de Braun e Clarke.

**Resultados:**

participaram oito profissionais, sendo seis enfermeiros e dois técnicos de enfermagem, com graus de perda auditiva de leve a profunda. Emergiram dois temas principais - Acessibilidades e Barreiras - e quatro subtemas: acessibilidades atitudinais (representatividade, apoio e convivência inclusiva) e comunicacionais (uso de estratégias adaptadas de comunicação); barreiras atitudinais (capacitismo, invisibilidade da surdez e discriminação) e comunicacionais (máscaras de proteção, ambientes ruidosos, uso da escrita e fadiga auditiva). Apesar de avanços pontuais, persistem barreiras estruturais e culturais que dificultam a plena inclusão desses profissionais.

**Conclusão:**

a inclusão de pessoas com deficiência auditiva na enfermagem ainda é parcial nas dimensões atitudinal e comunicacional. A presença desses profissionais contribui para a conscientização sobre a surdez, quebra de estigmas e fortalecimento de práticas de acessibilidade, essenciais para uma enfermagem mais diversa e equitativa.

## Destaques

**(1)** A inclusão de pessoas com deficiência auditiva na enfermagem ainda é parcial. **(2)** A presença de profissionais surdos contribui para a conscientização sobre a surdez. **(3)** Essa presença auxilia na redução das desigualdades no acesso à saúde. **(4)** Com as devidas adaptações, a comunicação torna-se viável. **(5)** É fundamental a colaboração entre a sociedade e os profissionais da enfermagem surdos.

## Introdução

Segundo a Pesquisa Nacional por Amostra de Domicílio (PNAD Contínua) de 2022, 1,2% da população total do País, ou seja, 2,51 milhões de brasileiros, possuem dificuldade para ouvir mesmo com uso de aparelho auditivo^1^. Entretanto, essa parcela populacional ainda encontra dificuldade de adentrar diversos espaços sociais, como o mercado de trabalho^1^. A PNAD também aponta que, dos indivíduos com dificuldade para ouvir, 24,4% possuem uma ocupação. De acordo com a Relação Anual de Informações Sociais (RAIS) de 2023, o número de pessoas com deficiência auditiva (DA) no mercado de trabalho formal era de 113.471 trabalhadores, totalizando 0,21% do estoque total do país^1^. 

Esse cenário de baixa inserção se repete na área da saúde, assim como para aqueles que desejam adentrar na enfermagem^2^. Além disso, não há sequer dados estatísticos disponíveis sobre o número total de profissionais da enfermagem com surdez nas apurações dos órgãos da classe. Por fim, de acordo com o Censo da Educação Superior, entre os anos de 2010 e 2023, dos alunos que se graduaram em enfermagem, somente 0,04% tinham DA e 0,01% tinham surdez^3^.

Dessa forma, pouco se sabe sobre a realidade desses profissionais, sobre como é atuar na enfermagem e conviver com a DA ao mesmo tempo. Visto isso, buscou-se explorar a inclusão de profissionais da enfermagem surdos nas dimensões da acessibilidade atitudinal e comunicacional no ambiente de trabalho.

## Método

### Desenho do estudo 

Trata-se de um estudo descritivo, transversal, de abordagem qualitativa^4^.

### Referencial teórico

A Inclusão Social é um movimento em prol de pessoas com deficiência (PCD) que iniciou no fim do século 20 e início do 21. Seu objetivo é instituir uma sociedade verdadeiramente para todos, baseando-se em seis princípios: celebração das diferenças, direito de pertencer, valorização da diversidade humana, solidariedade humanitária, igual importância das minorias e cidadania com qualidade de vida. Para isso, é necessária a cooperação entre sociedade e PCD^5^.

Entretanto, as práticas sociais em relação às PCD já atravessaram diferentes fases ao longo dos anos. Inicialmente, várias culturas praticavam a Exclusão Social de sujeitos com condições atípicas, os apartando, institucionalizando-os ou até eliminando-os, uma vez que esses eram considerados inválidos. Em seguida, passaram a surgir entidades especializadas por tipo de deficiência, como escolas especiais, centros de habilitação e reabilitação, oficinas protegidas de trabalho, entre outros, dando início à Segregação Institucional. A partir de 1960, surgiu a Integração Social, na qual não se objetivava uma plena participação de indivíduos com deficiência nos setores sociais, mas simplesmente uma inserção deles nos espaços de convívio sem que adaptações adequadas fossem feitas. As PCD que passassem a participar ativamente da sociedade o fariam por meio de mérito pessoal, alcançando os padrões sociais definidos^5^.

Para Sassaki, existem sete dimensões da acessibilidade, elaboradas de forma a esclarecer a aplicação prática dos conceitos propostos no movimento da inclusão, auxiliando na identificação das barreiras de acessibilidade e mitigação dessas^6^. São elas: arquitetônica, atitudinal, comunicacional, instrumental, metodológica, natural e programática. Nesta investigação, destacam-se as dimensões atitudinal e comunicacional nos espaços de trabalho. A primeira versa sobre acesso sem barreiras resultantes de preconceitos, estigmas, estereótipos e discriminações que impeçam ou prejudiquem a participação social da pessoa com deficiência em igualdade com os demais^6^ ^-^ ^7^. Já a segunda versa sobre o acesso sem barreiras na comunicação em suas mais diferentes formas: escrita, verbal, gestual, Língua Brasileira de Sinais (Libras), facial, corporal, pessoalmente ou à distância, na saúde, e entre outros^5^. 

### Local e período da coleta de dados

A coleta de dados ocorreu por meio de entrevistas via *Zoom Meeting*, de 15 de agosto a 11 de dezembro de 2024. Durante as entrevistas, tanto os participantes como o entrevistador estavam em suas casas, em um cômodo com privacidade. 

### Participantes

Os participantes foram pessoas com surdez, com idade ≥ 18 anos, profissionais de enfermagem (enfermeiro, técnico ou auxiliar) que possuíam inscrição ativa em conselho de classe e que estavam atuando em qualquer área relacionada à enfermagem. A técnica de amostragem utilizada foi a bola de neve^4^. 

### Instrumento de coleta de dados 

Optou-se por realizar entrevistas semiestruturadas via *Zoom Meeting,* conduzidas pela primeira autora, enquanto aluna do mestrado profissional em enfermagem, com orientação da segunda autora, enquanto orientadora. Foi realizada uma entrevista-piloto, visto que esta era a primeira experiência da aluna, para testagem e possíveis ajustes. 

A duração média das entrevistas foi de 40 minutos, incluindo a apresentação do entrevistador (nome e instituição), a explicação dos objetivos, o esclarecimento de dúvidas, a resposta às perguntas e o encerramento. As conversas foram gravadas em vídeo e áudio utilizando o recurso da própria plataforma. Também foram tomadas notas das entrevistas, como impressões do entrevistador. 

O roteiro das entrevistas foi elaborado em duas partes: caracterização demográfica e perguntas norteadoras (Figura 1).

**Figura 1:** Roteiro da entrevista

Perguntas	Alternativas	
Qual sua categoria profissional?	Enfermeiro	Técnico de enfermagem
	Auxiliar de enfermagem	
Qual seu grau de surdez?	Leve	Moderado
	Severo	Profundo
Quando sua surdez se instalou?	Pré-lingual	Pós-lingual
Você é oralizado?	Sim	Não
Qual sua primeira língua?	Português	Libras
Você sabe Libras?	Sim	Não
Você utiliza aparelho auditivo?	Sim	Não
Você faz leitura labial?	Sim	Não
Idade:		
Cidade onde você trabalha:		
Ano de formação no curso técnico ou graduação:		
Tempo de atuação na enfermagem:		
Você trabalha em qual nível de atenção à saúde?	Nível primário	Nível secundário
	Nível terciário	
Seu trabalho é da iniciativa:	Pública	Privada
Como foi para você adentrar o mercado de trabalho sendo uma pessoa com surdez?		
Foi feita alguma adaptação para receber você onde você trabalhou/trabalha?		
Você considera que existem pontos positivos em ser um profissional da enfermagem com surdez? Se sim, quais?		
Você considera que existem desafios na atuação enquanto profissional da enfermagem com surdez? Se sim, quais?		

As entrevistas foram realizadas apenas uma vez com cada participante em data e hora de sua preferência. Apenas um participante, por se comunicar exclusivamente por meio de Libras, necessitou do acompanhamento de um intérprete. 

### Coleta de dados 

Todo o banco de dados foi armazenado na plataforma *Research Electronic Data Capture* (REDCap). 

Os intermediários iniciais, ou “sementes”, foram indivíduos com quem a pesquisadora já possuía contato prévio e que, inclusive, poderiam tornar-se participantes^4^. Para que pudessem convidar outros participantes, as sementes receberam uma mensagem específica sobre o projeto e um link para o preenchimento do Termo de Consentimento Livre e Esclarecido (TCLE). Após o aceite, o participante indicou três dias e horários para a realização da entrevista e poderia apontar se haveria necessidade de intérprete de Libras durante a conversa. 

Foi então solicitado que aqueles que aceitaram participar da pesquisa convidassem outras pessoas^4^. O encerramento da coleta foi definido quando não surgiram novos participantes, e entendeu-se que os dados obtidos já permitiam alcançar o objetivo proposto.

Foram registrados 17 acessos ao REDCap, mas apenas oito resultaram em entrevistas válidas. Oito acessos estavam incompletos a partir do TCLE e um participante foi excluído por não ser graduado em enfermagem, sendo orientado sobre os critérios de inclusão.

### Análise dos dados

Primeiramente, as gravações das entrevistas foram anexadas ao *software* Transkriptor, que utiliza Inteligência Artificial (IA) para realizar a transcrição de áudios em textos^8^. Contudo, para garantir a qualidade dos dados, todo material foi revisado diversas vezes para corrigir quaisquer falhas. As transcrições não foram devolvidas para os participantes. 

Em conformidade com o conceito de Ciência Aberta, todo conjunto de dados deste artigo, isto é, as entrevistas na íntegra, está disponível no repositório SciELO Data sob o mesmo nome deste artigo, por meio do link https://doi.org/10.48331/scielodata.0FSQFM.

A análise dos dados foi feita segundo a análise temática de Braun e Clarke. Esse processo foi feito dentro da plataforma Taguette, uma ferramenta gratuita, que facilita na codificação, categorização, identificação de padrões e geração de relatórios^9^. 

A familiarização com o banco de dados ocorreu durante a transcrição das entrevistas, seguida de leitura integral do material. As fases dois e três envolveram a criação de códigos e temas iniciais, com foco nas individualidades de cada entrevista. Na fase quatro, realizou-se uma nova análise para refinamento das categorias, considerando o conjunto das entrevistas. Observou-se então a relação das categorias com as sete dimensões da acessibilidade propostas por Sassaki, o que levou a uma nova análise para verificar a adequação dos achados a essas dimensões^10^.

### Considerações éticas

O estudo foi conduzido de acordo com as diretrizes de ética nacionais e internacionais e aprovado pelo Comitê de Ética em Pesquisa do Hospital Israelita Albert Einstein, CAAE: 77221424.0.0000.0071, n.º do parecer 6.885.669. 

Os participantes foram identificados por meio de um código alfanumérico (p. ex. P1, P2...), omitindo-se quaisquer informações sensíveis das transcrições.

## Resultados

Ao final, foram realizadas oito entrevistas. As idades dos participantes variaram entre 26 e 39 anos, com média de 31 anos. Seis participantes são enfermeiros e dois são técnicos de enfermagem. 

Os graus de surdez relatados variaram de leve a profundo, sendo que apenas três adquiriram a condição antes de aprender a falar. Apenas um participante possuía a Libras como primeira língua e não o português. Este mesmo indivíduo era o único que se comunicava somente pela Língua Brasileira de Sinais. 

Dois participantes informaram saber Libras, três relataram saber somente o básico e três não possuíam nenhum conhecimento deste idioma. Somente dois indivíduos não utilizavam aparelho auditivo, os outros cinco utilizavam em ambos os ouvidos. Todos os entrevistados declararam fazer leitura labial, empregando esse recurso com pouca ou grande frequência, conforme suas necessidades. 

Quanto à carreira dos participantes na enfermagem, o tempo de atuação variou de oito meses a 17 anos, com média de 6,8 anos. Quatro atuavam no setor terciário de atenção à saúde e um no secundário. Para três participantes, essa pergunta não se aplicou, pois um trabalhava em um segmento governamental da saúde e dois eram professores. Por fim, os participantes eram das regiões Nordeste, Sul e Sudeste do Brasil.

Ao todo, a análise passou por três versões até que fosse atingido um resultado satisfatório. Das sete dimensões da acessibilidade^6^, somente cinco eram aplicáveis aos resultados obtidos nas entrevistas. Entretanto, o presente artigo objetiva discutir somente as dimensões atitudinal e comunicacional. Estas dimensões foram então divididas em acessibilidades e barreiras. É fundamental ressaltar que os temas foram construídos a partir dos resultados obtidos e não previamente estabelecidos. Além disso, as descrições finais destes foram baseadas nos conceitos apresentados por Sassaki e adaptadas conforme as necessidades da análise. 

Os temas são Acessibilidades e Barreiras. O primeiro diz respeito aos relatos nos quais foi possível identificar contextos alinhados a esforços de inclusão. Já o segundo é relativo aos relatos em que foi possível identificar barreiras que dificultaram a inclusão. 

Do primeiro tema derivam os subtemas Acessibilidades atitudinais, com 34 códigos, e Acessibilidades comunicacionais, com 40 códigos. Do tema Barreiras derivam os subtemas Barreiras atitudinais, com 19 códigos, e Barreiras comunicacionais, com 24 códigos. 

### Acessibilidades atitudinais

Demonstram atitudes e comportamentos favoráveis à inclusão, como representatividade, apoio e convivência em igualdade. Alguns exemplos de códigos são:

*No final, eu disse “[...] eu sou enfermeira dele, mas que eu também sou deficiente auditiva.” [...]. Os avós daquela criança chegaram e disseram, “A DA não vai impedir o meu neto de ser quem ele quiser.” (P6)*

*[...] eu acho que quando a pessoa quer e tem outras pessoas que ajudam e que adaptam, sempre é possível. (P5)*

*Muitas vezes, as meninas [equipe de enfermagem] falavam para mim que só depois que tiveram contato comigo que elas souberam como elas agiriam com esses pacientes [com DA], com a família desses pacientes. (P6)*

*[...] hoje todos os meus pacientes, quando eu falo, “Eu sou deficiente auditiva [...]”, todos eles super aceitaram. (P6)*

*Eu acredito que eu não teria o nível de empatia que eu tenho hoje se eu não tivesse tido essa condição. (P1)*

### Acessibilidades comunicacionais

Demonstram o estabelecimento da comunicação entre o participante e outros profissionais, pacientes e suas famílias, por meio da adoção de diferentes estratégias. Alguns exemplos de códigos são:

*[...] quando eu cheguei no setor, eu tive que dizer para todo mundo [...] “Eu não escuto bem, se você quiser falar comigo, fala comigo de frente ou toca em mim, que aí eu consigo falar com você, porque se você falar comigo de costas, eu não vou conseguir entender.” (P1)*

*[...] eu falei assim, “Olha, já que você acha que a minha DA está impactando assim, então, a partir de agora, eu solicito que você me passe todo o plantão de todos os pacientes por escrito.” (P5)*

*Eu fui atender um paciente e perguntei se ele poderia tirar a máscara, ficar só com a viseira [protetor facial transparente] e a gente manter a distância. Ele ficou com medo, mas tirou. (P2)*

*Quando eu vou me comunicar com pessoas idosas, eu uso mais gestos, porque eles já são idosos e aí não conseguem ler por conta da visão. (P7)*

### Barreiras atitudinais

Demonstram atitudes e comportamentos desfavoráveis à inclusão, como capacitismo, invisibilidade da surdez e discriminação. Alguns exemplos de códigos são:

*E no âmbito do trabalho também, porque infelizmente há um capacitismo que as pessoas acham que, porque você é pessoa com uma deficiência, você é incapaz ou menos. [...] Não tem tanta habilidade como alguém ouvinte. (P1)*

*Acho que precisa ter mais abrangência na hora de receber a gente, e não menosprezar, porque apesar de não parecer, eu ainda sou um deficiente. (P3)*

*Eu soube [...] que houve colegas que perguntaram se eu podia ir para outro setor porque eu não ia conseguir estar naquele setor sendo que eu era uma pessoa que não escuta. (P1)*

*Então eu tive algumas dificuldades, realmente, com essas questões [compreensão dos outros profissionais da saúde quanto a DA] e eu pedi ajuda da coordenação. Foi bem atendido? Não necessariamente. (P3)*

*[...] teve uma paciente que queria aferir a pressão [...] e ela falou, “Nossa, só tem uma enfermeira no ambulatório e ainda me põe uma surda?”. (P5)*

*Militar sobre isso é cansativo, muito cansativo. (P2)*

### Barreiras comunicacionais

Demonstram barreiras comunicacionais ocasionadas ou agravadas pela DA, como o uso de máscaras de proteção, ambientes ruidosos, uso da escrita e fadiga auditiva. Alguns exemplos de códigos são:

*[...] é um ambiente [hospital] que estava todo mundo de máscara, então o meu poder de ler lábios acabou ali. (P3)*

*Aparelho auditivo nunca vai ser um ouvido. O aparelho aumenta muito todos os sons, todos! Então o barulho do ar-condicionado, para mim, vai ser muito alto, mas o barulho da voz, para mim, não vai ser alto. (P3)*

*[...] eu vejo que ainda tem uma certa barreira. Por exemplo, eu trabalho dentro da hemodinâmica, e a gente sabe que [...] tem barulhos. Tem alarme de bomba, alarme de monitor, alarme do cardioversor [...]. (P6)*

*Além disso, você sabe que surdos escrevem, muitas vezes, um Português de estrangeiro. Não é o Português convencional como todo mundo escreve. (P7)*

*[...] uma pessoa que não escuta bem e é profissional da saúde, lamentavelmente, já é uma pessoa muito mais ansiosa e cansada. [...] porque a gente faz um esforço maior do que as outras pessoas para entender as informações [...]. (P4)*

*O meu desafio maior é trabalhar nos setores mais confusos, tipo UTI e emergência, áreas que têm muito movimento, onde as pessoas não têm tempo de parar, muitas vezes, para conversar comigo, para dar atenção. (P7)*

## Discussão

A discussão dos temas foi organizada por dimensão. Inicialmente, serão apresentadas as formas de acessibilidade observadas, seguidas das respectivas barreiras em cada uma delas. Essa estrutura permite comparar os dois cenários de forma direta, possibilitando uma visualização mais clara e integrada de cada dimensão analisada.

### Acessibilidades atitudinais

Um tema frequente nos relatos foram cenários de representatividade. Também conhecida como representação identitária, consiste em pessoas que atuam como referência de uma população, visto que possuem características similares às dos indivíduos representados^11^. A presença desses indivíduos nas esferas sociais aumenta a conscientização da sociedade sobre PCD e a inclusão, como indicado na interação do P6 com a família de uma criança. 

Por vezes, pacientes são afligidos por condições que podem ser vividas pelos próprios profissionais que lhes ofertam cuidado. Ou seja, a inclusão e a diversidade na saúde trazem ganhos substanciais no cuidado e na experiência do paciente^12^. Importa destacar, ainda, que a presença de profissionais cuja língua e cultura sejam iguais às de seus pacientes colabora com a redução das desigualdades de acesso à saúde. Adicionalmente, auxilia nesse processo por promover a educação e a conscientização dos demais profissionais sobre PCD^13^. 

Outro ponto constantemente destacado foi a importância e o valor de receber apoio dos demais profissionais e das coordenações das instituições. Esse apoio, especialmente quando feito de forma genuína e não por considerar o indivíduo incapaz ou por pena, promove no profissional com deficiência maior autoconfiança e estabilidade^14^. Sassaki afirma que uma das consequências da inclusão é que este processo acaba por beneficiar não somente ao indivíduo com deficiência, mas também a comunidade^5^ ^-^ ^6^, como exemplificado pelos participantes 5 e 6.

Quanto aos pacientes e à aceitação deles em receber cuidados ofertados por um profissional da saúde com DA, embora existam relatos de recepção negativa, a maioria dos entrevistados relatou cenários positivos, como exemplificado por P6. Em uma investigação composta por profissionais da saúde com DA de diferentes áreas, evidenciou-se que, geralmente, os pacientes demonstravam reações neutras ou positivas ao atendimento desses profissionais^12^. 

Além disso, uma característica que se relaciona com o cuidado ofertado é a empatia do profissional com seus pacientes. Um estudo demonstrou que estudantes de medicina com diferentes deficiências tinham menores chances de terem baixos níveis de empatia^15^, característica apontada pelo P1 como um ponto positivo que a surdez trouxe para sua atuação na enfermagem. Sendo assim, os achados de ambos os estudos estão em concordância com os resultados obtidos nesta investigação.

### Barreiras atitudinais

Qualquer indivíduo pode enfrentar desafios no mercado de trabalho. Entretanto, aqueles com deficiência experienciam o *minority tax*, que, em tradução livre, seria “taxa para minorias”. Isso significa que, além das responsabilidades e dificuldades naturais desse ambiente, PCD lidam com entraves adicionais devido ao preconceito, isolamento, exclusão, entre outros^12^. 

Um dos componentes desse fenômeno é o capacitismo. Este termo refere-se a julgar e discriminar uma pessoa com base nas capacidades corporais e mentais dela e usar isso para classificá-la como incapaz^2^. Infelizmente, alguns entrevistados, assim como P1, relataram ter experienciado esse fenômeno. É importante ressaltar que se trata de um constructo estrutural da sociedade, resultado de um sistema histórico de políticas, instituições e normas sociais, que desvalorizam e marginalizam PCD^2^. Os relatos indicam que ele pode partir de iniciativa institucional, como, por exemplo, não fornecendo acessibilidade comunicacional, ou pode ser em âmbito individual, mediante atitudes negativas como, por exemplo, utilizando linguagem discriminatória. 

Estudos sobre discriminação e capacitismo sofridos por profissionais da saúde com deficiência comprovam que este cenário é recorrente. Além disso, pode desencorajar estas pessoas a atuarem no sistema de saúde, ou até impactar seu bem-estar^2^. Felizmente, no caso dos participantes desta pesquisa, embora tenha gerado certo aborrecimento, não houve maiores complicações oriundas de atitudes capacitistas. 

Outro fator relevante, é a invisibilidade da DA. Não se pode distinguir um indivíduo com DA das demais pessoas, a menos que este decida revelar esta informação. Por vezes, o uso de implantes auditivos é um dos únicos meios de tornar visível esta característica^16^. Um dos entrevistados (P3) relatou que, por conta da invisibilidade da surdez, outras pessoas tiveram uma percepção equivocada de que a DA gera mínimos impactos em sua vida profissional. 

Esse relato evidencia que a invisibilidade da surdez pode gerar formas sutis de negação da deficiência, reforçando o capacitismo e criando obstáculos adicionais para a plena inclusão de PCD auditiva na sociedade.

Outro entrave, componente do *minority tax*, e um dos mais importantes, é a discriminação. Vários relatos sobre discriminação foram reportados sobre diversas interações, com outros profissionais da saúde (P1), coordenação das instituições (P3), pacientes e suas famílias (P5). 

Esses relatos são um reflexo da falta de PCD nos espaços sociais e da ignorância das pessoas quanto a estes indivíduos. Existem diversos estereótipos sobre as capacidades de um surdo, como ser menos inteligente, ter menos capacidade comunicacional, não ter habilidades sociais, não ter capacidade para escrever, entre outros^12^. Sassaki propõe que um dos principais meios para lidar com a discriminação é a educação quanto à diversidade e a inclusão de PCD em todas as esferas da sociedade. Uma das recomendações são programas de sensibilização destinados a funcionários, que têm demonstrado resultados positivos na redução de barreiras atitudinais no ambiente de trabalho^6^. 

Por fim, é fundamental ressaltar que esta conscientização precisa ser feita a partir da iniciativa das organizações, uma vez que, frequentemente, cabe somente ao indivíduo com deficiência fazer essa educação *in loco*, advogando por si mesmo^6^ ^,^ ^12^. A necessidade de realizar autoadvocacia é um dos componentes do *minority tax* e, por ser uma demanda constante, torna-se exaustiva^12^, como mencionado pelo P2. Nesse sentido, vale relembrar que a inclusão verdadeira requer a colaboração e esforço mútuo entre PCD e sociedade^5^.

### Acessibilidades comunicacionais 

Um eixo temático importante identificado foi a incorporação de estratégias comunicacionais por parte das outras pessoas, conforme as necessidades específicas expressadas por cada participante, como exemplificado pelo P1. É possível identificar que, em determinadas situações, pequenas ações e de fácil implementação, como posicionar-se de frente ao conversar com uma pessoa surda, podem tornar a comunicação acessível. Entretanto, cabe a ressalva de que observou-se novamente que recai sobre as PCD a necessidade de conscientizar e instruir as demais pessoas sobre os métodos que existem para gerar inclusão, visto que muitos não possuem esse conhecimento^5^ ^,^ ^12^. Reforçando, portanto, a assimetria no empenho feito para que exista inclusão, assim como descrito por Sassaki em suas análises sobre práticas integrativas^5^ ^-^ ^6^. 

Uma especificidade da rotina de profissionais de enfermagem é a passagem de plantão, que geralmente é feita verbalmente. Entretanto, foi feita uma adequação na forma de transmissão das informações após o entrevistado P5 relatar ter dificuldades com esse processo, visto que o cenário era de múltiplas pessoas falando ao mesmo tempo. 

Este exemplo reafirma o que foi apresentado em um estudo sobre pessoas com perda auditiva unilateral, que ressaltou que ambientes ruidosos prejudicam a capacidade do indivíduo de compreensão da fala^17^ ^-^ ^18^. Além disso, em um cenário como o descrito pelo participante, com múltiplas pessoas falando ao mesmo tempo, fazer a leitura labial de todos não é possível. Sendo assim, mudanças como a mencionada podem trazer mais segurança para o profissional e para o paciente, visto que uma comunicação efetiva é crucial para o cuidado adequado^19^. 

Por fim, os entrevistados relataram algumas estratégias que desenvolveram para estabelecer comunicações efetivas com seus pacientes, como, por exemplo, a leitura labial e gestos no caso do P2 e P7, respectivamente. Estudos apresentam outras estratégias utilizadas por profissionais da saúde com surdez para estabelecer comunicação com seus pacientes. Alguns exemplos são: sistemas de microfones, máscaras inclusivas mesmo que não padronizadas, intérpretes, cartões explicativos e aplicativos de transcrição simultânea de texto^19^. 

É possível inferir que a escolha da estratégia utilizada depende de múltiplos fatores, como disponibilidade dos recursos, necessidades do profissional com DA e especificidades de cada paciente. Desta forma, não há como estabelecer um método ideal padronizado. Sendo assim, devem estar disponíveis diversas formas de comunicação, para que o profissional possa escolher as que melhor se aplicam a cada caso. 

### Barreiras comunicacionais

Diversos ambientes de atuação da enfermagem envolvem o uso da máscara de proteção na maior parte do tempo. Durante a pandemia da Covid-19, esse uso tornou-se ainda mais necessário, e ocorreu em situações nas quais, no dia a dia, não era corriqueiro, gerando impactos significativos para PCD auditiva^20^. 

Como exemplificado por P6, o uso da máscara impacta na habilidade do indivíduo de fazer leitura labial, que muitas vezes é um complemento da comunicação. No caso de surdos que utilizam Libras, o uso da máscara impede a percepção das expressões faciais, que são importantes na língua de sinais^20^. Dessa forma, o uso das máscaras gerou maiores níveis de estresse e ansiedade em profissionais da saúde com DA. Alguns participantes ressaltaram que isso os deixou receosos com o futuro na carreira, levando-os a questionar se precisariam mudar de profissão, uma vez que essa barreira parecia impossível de ser vencida. Portanto, essa barreira também prejudicou o senso de autonomia, independência e pertencimento desses profissionais, favorecendo sensação de isolamento e incapacidade de demonstrar competência profissional^20^.

Outro fator que prejudica a comunicação é a percepção auditiva em ambientes ruidosos. Alguns exemplos onde isso ocorre, segundo os participantes, seriam centros cirúrgicos, emergências, UTIs, hemodinâmicas, entre outros. Frequentemente, indivíduos em uso de próteses auditivas referem ter dificuldade com a discriminação auditiva da fala em ambientes acusticamente desafiadores. Nestas instâncias, cabe ao indivíduo dispender maior esforço cognitivo para discernir a fala dos demais ruídos. Este esforço requer uma carga de concentração alta, que deixa de ser destinada a outras atividades^21^. 

Quanto à utilização da escrita como estratégia comunicacional, é importante ressaltar que, embora possa auxiliar em algumas situações, este método pode não ser totalmente efetivo. Aqueles indivíduos com surdez que têm o português escrito como segunda língua podem apresentar dificuldades com esta modalidade de comunicação, devido às diferenças semânticas e gramaticais da Libras e do português^19^. No caso de indivíduos com DA que tenham o português como primeira língua, é posto que estes podem apresentar dificuldades no aprendizado da língua, falada e escrita, em decorrência da DA^17^ ^-^ ^18^. Em vista disso, a comunicação por escrito não deve ser considerada como meio principal de estabelecer uma comunicação efetiva com um indivíduo com surdez^19^.

Considerando todas essas barreiras comunicacionais, participantes como P4 relataram sentir cansaço mental resultante do esforço contínuo para conseguir se comunicar. A fadiga auditiva também é relatada por outros indivíduos com surdez após estarem em ambientes ruidosos, que demandam interatividade e, portanto, maior carga cognitiva e de concentração. Como resultado, pode levar a maiores níveis de estresse^17^ ^-^ ^18^. Entretanto, este cansaço mental não foi a única consequência identificada nas barreiras comunicacionais. Foi relatado por participantes como P7 que determinadas áreas de atuação da enfermagem passaram a ser consideradas como inviáveis para profissionais com DA, devido às barreiras encontradas, especialmente as de comunicação.

 Este relato exemplifica a fase de integração, uma vez que pessoas com DA estão adentrando ambientes sem receber as adaptações necessárias. Desse modo, estes indivíduos precisam se adaptar como podem^4^. É comum que, mesmo que de maneira não intencional, instituições acabem desencorajando estes indivíduos a atuarem em subespecialidades. Isso se deve tanto por não fornecerem as adaptações necessárias, como também pela própria crença de que estes indivíduos não deveriam ou não teriam capacidade para estar em espaços de contato com o paciente, ou onde a comunicação deve ser rápida. Contudo, com os recursos necessários, é possível que pessoas com DA desempenhem sua profissão com qualidade e segurança^12^.

Este estudo apresentou como limitação um número de participantes inferior ao esperado, visto que se tinha como estimativa 15 indivíduos. Entretanto, esta dificuldade já era prevista dado as limitações de acesso a essa população^22^. De qualquer forma, acredita-se que foi possível obter resultados significativos sobre a temática e trazer um olhar inovador sobre a atuação de profissionais da enfermagem surdos, visto que qualquer proposta feita sobre PCD deve envolver as próprias. 

## Conclusão

Ao comparar o conceito de inclusão apresentado por Sassaki e as experiências dos participantes, é possível compreender que, nas dimensões de acessibilidade atitudinal e comunicacional, a inclusão plena de pessoas com DA na enfermagem ainda está distante de ser concretizada. A ignorância da sociedade sobre PCD e os meios que existem para mitigar barreiras configuram-se como uma das principais causas dos desafios identificados. 

Entretanto, relatos positivos demonstram que esta é uma realidade em construção. Para isso, vale ressaltar que é imperativo que a sociedade assuma um papel mais ativo no processo de inclusão de PCD, de forma que não recaia somente sobre elas.

Por outro lado, a presença de PCD auditiva na enfermagem contribui para a conscientização da sociedade sobre PCD, mostrando que a deficiência não impede a atuação na enfermagem, quebrando estigmas e preconceitos e reforçando a importância da implementação de estratégias de acessibilidade. Por consequência, essa presença auxilia na redução das desigualdades no acesso à saúde para outras pessoas com deficiência. Na dimensão comunicacional, é possível compreender que, com as devidas adaptações, a comunicação torna-se viável para os profissionais desempenharem suas atribuições com segurança, autonomia e menores níveis de esforço mental. 

## Data Availability

O conjunto de dados deste artigo está disponível na página da RLAE no repositório SciELO Data, no link https://doi.org/10.48331/scielodata.0FSQFM.

# Translation: Profesionales de enfermería sordos: experiencias de interacción social y comunicación en la práctica profesional^*^

**DOI:** 10.1590/1518-8345.8211.4911

**Authors:** Aline de Barros Campanelli Lima, Bárbara Barrionuevo Bonini

**Affiliations:** 1 Instituto Israelita de Ensino e Pesquisa Albert Einstein, Unidade Paulista II, São Paulo, SP, Brasil.

**Keywords:** Sordera, Enfermería, Inclusión Social, Práctica Profesional, Interacción Social, Comunicación

## Abstract

**Objetivo:**

explorar la inclusión de profesionales de la enfermería sordos en las dimensiones de la accesibilidad actitudinal y comunicacional en el entorno laboral.

**Método:**

un estudio cualitativo, transversal y descriptivo, basado en la historia de la inclusión de personas sordas y en las siete dimensiones de la accesibilidad de Sassaki. Se realizaron entrevistas semiestructuradas online con profesionales de la enfermería sordos. El análisis siguió el método de Análisis Temático de Braun y Clarke.

**Resultados:**

participaron ocho profesionales, seis enfermeras y dos técnicos de enfermería, con grados de pérdida auditiva que iban de leve a profundo. Surgieron dos temas principales - Accesibilidades y Barreras - y cuatro subtemas: accesibilidades actitudinales (representatividad, apoyo y convivencia inclusiva) y de comunicación (uso de estrategias de comunicación adaptadas); barreras actitudinales (capacitismo, invisibilidad de la sordera y discriminación) y de comunicación (mascarillas protectoras, ambientes ruidosos, uso de la escritura y fatiga auditiva). A pesar de los avances ocasionales, persisten barreras estructurales y culturales que dificultan la plena inclusión de estos profesionales.

**Conclusión:**

la inclusión de personas sordas en enfermería sigue siendo parcial en las dimensiones actitudinal y comunicativa. La presencia de estos profesionales contribuye a aumentar la concienciación sobre la sordera, romper estigmas y fortalecer las prácticas de accesibilidad, esenciales para una enfermería más diversa y equitativa.

## Destacados

**(1)** La inclusión de personas sordas en la enfermería sigue siendo parcial. **(2)** La presencia de profesionales sordos contribuye para la concientización sobre la sordera. **(3)** Esta presencia ayuda a reducir las desigualdades en el acceso a la salud. **(4)** Con las adaptaciones adecuadas, la comunicación se vuelve factible. **(5)** La colaboración entre la sociedad y los profesionales de la enfermería sordos es esencial.

## Introducción

Según la *Pesquisa Nacional por Amostra de Domicílio* (Encuesta Nacional Continua por Muestreo de Hogares) de 2022 (*PNAD Contínua*), el 1,2% de la población total del país, es decir, 2,51 millones de brasileños, tiene dificultades auditivas incluso usando audífonos^1^. Sin embargo, esta parte de la población aún encuentra dificultades para acceder a diversos espacios sociales, como el mercado laboral^1^. La PNAD también señala que, de las personas con dificultad auditiva, el 24,4% tiene una ocupación. Según la Relación Anual de Informaciones Sociales 2023 (RAIS), el número de personas con dificultad auditiva (DA) en el mercado laboral formal fue de 113.471 trabajadores, lo que supone un total del 0,21% del total del país^1^.

Este escenario de baja inserción se repite tanto en el área de salud, como en quienes desean acceder a la enfermería^2^. Además, ni siquiera existen datos estadísticos disponibles sobre el número total de profesionales de enfermería con sordera en los cálculos de los cuerpos de clase. Finalmente, según el Censo de Educación Superior, entre 2010 y 2023, de los estudiantes que se graduaron en enfermería, solo el 0,04% tenían DA y el 0,01% sordera^3^.

Por tanto, se sabe poco sobre la realidad de estos profesionales, sobre cómo es trabajar en enfermería y vivir con la DA al mismo tiempo. Por ello, buscamos explorar la inclusión de profesionales de enfermería sorda en las dimensiones de accesibilidad actitudinal y comunicativa en el entorno laboral.

## Método

### Diseño del estudio 

Se trata de un estudio descriptivo y transversal con un enfoque cualitativo^4^.

### Marco teórico

La Inclusión Social es un movimiento a favor de las personas con discapacidad (PCD) que comenzó a finales del siglo XX y principios del XXI. Su objetivo es establecer una sociedad verdaderamente para todos, basada en seis principios: celebración de las diferencias, derecho a pertenecer, apreciación de la diversidad humana, solidaridad humanitaria, igualdad de importancia de las minorías y ciudadanía con calidad de vida. Para ello, es necesaria la cooperación entre la sociedad y las PCD^5^.

Sin embargo, las prácticas sociales relacionadas con las PCD han pasado por diferentes fases a lo largo de los años. Inicialmente, varias culturas practicaban la Exclusión Social de sujetos con condiciones atípicas, separándolos, institucionalizándolos o incluso eliminándolos, ya que se consideraban inválidos. Posteriormente, comenzaron a surgir entidades especializadas por tipo de discapacidad, como escuelas especiales, centros de habilitación y rehabilitación, talleres de trabajo protegido, entre otros, iniciando la segregación institucional. A partir de 1960 surgió la Integración Social, cuyo objetivo no era la participación plena de las PCD en los sectores sociales, sino simplemente su inserción en espacios sociales sin realizar adaptaciones adecuadas. Las PCD que empezaron a participar activamente en la sociedad lo harían por mérito personal, alcanzando los estándares sociales definidos^5^.

Para Sassaki, existen siete dimensiones de accesibilidad, elaboradas para clarificar la aplicación práctica de los conceptos propuestos en el movimiento de inclusión, ayudando a identificar y mitigar las barreras de accesibilidad^6^. Son: arquitectónica, actitudinal, comunicacional, instrumental, metodológica, natural y programática. En esta investigación, se destacan las dimensiones actitudinales y comunicativas en los espacios de trabajo. La primera trata sobre el acceso sin barreras derivadas de prejuicios, estigmas, estereotipos y discriminación que impiden o perjudican la participación social de las PCD en igualdad de condiciones con las demás^6^ ^-^ ^7^. La segunda trata sobre el acceso sin barreras a la comunicación en sus formas más diferentes: escrita, verbal, gestual, Lengua Brasileña de Señas (Libras), facial, corporal, en persona o a distancia, en salud, entre otras^5^.

### Lugar y periodo de la recogida de datos

La recogida de datos se realizó mediante entrevistas a través del *software Zoom*, del 15 de agosto al 11 de diciembre de 2024. Durante las entrevistas, tanto los participantes como el entrevistador estaban en sus casas, en una habitación con privacidad.

### Participantes

Los participantes eran personas con sordera, de ≥ 18 años, profesionales de enfermería (enfermera, técnica o asistente) que estaban activamente inscritos en los consejos de clase y que trabajaban en cualquier área relacionada con la enfermería. La técnica de muestreo utilizada fue bola de nieve^4^.

### Instrumento de recogida de datos 

Se decidió realizar entrevistas semiestructuradas vía Zoom, realizadas por el primer autor, como estudiante del máster profesional en enfermería, con la orientación del segundo autor, como asesor. Se realizó una entrevista piloto, ya que era la primera experiencia del estudiante, para realizar pruebas y posibles ajustes.

La duración media de las entrevistas fue de cuarenta minutos, incluyendo la introducción del entrevistador (nombre e institución), la explicación de los objetivos, la aclaración de dudas, la respuesta a las preguntas y el cierre. Las conversaciones se grabaron en vídeo y audio utilizando el propio recurso de la plataforma. También se tomaron notas de las entrevistas, como impresiones del entrevistador.

El guion de la entrevista se elaboró en dos partes: caracterización demográfica y preguntas orientadoras (Figura 1).

**Figura 1:** Guion de la entrevista

Preguntas	Alternativas	
¿Cuál es tu categoría profesional	Enfermero	Técnico de enfermería
	Auxiliar de enfermería	
¿Cuál es tu grado de sordera?	Leve	Moderado
	Severo	Profundo
¿Cuándo empezó tu sordera?	Pre lingual	Post lingual
¿Eres oral?	Sí	No
¿Cuál es tu lengua materna?	Portugués	Libras
¿Sabes Libras?	Sí	No
¿Usas audífonos?	Sí	No
¿Lees los labios?	Sí	No
Edad:		
Ciudad donde trabajas:		
Año de formación en el curso técnico o graduación:		
Tiempo trabajando en enfermería:		
¿A qué nivel de atención a la salud trabajas?	Nivel primario	Nivel secundario
	Nivel terciario	
Su trabajo proviene de la iniciativa:	Pública	Privada
¿Cómo fue para ti entrar en el mercado laboral siendo sorda?		
¿Se hizo alguna adaptación para recibirte donde trabajas/trabajas?		
¿Crees que hay aspectos positivos en ser un profesional de enfermería con sordera? Si es así, ¿cuáles?		
¿Crees que hay desafíos al trabajar como profesional de enfermería con sordera? Si es así, ¿cuáles?		

Las entrevistas se realizaron solo una vez con cada participante, en una fecha y hora de su preferencia. Solo un participante, ya que se comunica exclusivamente a través de Libras, necesitó el acompañamiento de un intérprete.

### Recogida de datos

Toda la base de datos se almacenó en la plataforma *Research Electronic Data Capture* (REDCap).

Los intermediarios iniciales, o “semillas”, eran individuos con los que el investigador ya había tenido contacto previo y que incluso podían llegar a ser participantes^4^. Para invitar a otros participantes, las semillas recibieron un mensaje específico sobre el proyecto y un enlace para rellenar el Formulario de Consentimiento Informado (CLPI). Tras la aceptación, el participante indicó tres días y horarios para la entrevista y pudo indicar si sería necesario un intérprete de Libras durante la conversación.

A quienes aceptaron participar en la investigación se les pidió entonces que invitaran a otras personas^4^. El final de la colección se definió cuando no aparecieron nuevos participantes, y se entendió que los datos obtenidos ya permitían alcanzar el objetivo propuesto.

Se registraron 17 accesos a REDCap, pero solo ocho resultaron en entrevistas válidas. Ocho admisiones fueron incompletas del CLPI y un participante fue excluido porque no tenía un título en enfermería, siendo instruido sobre los criterios de inclusión.

### Análisis de los datos

En primer lugar, las grabaciones de las entrevistas se adjuntaron al *software* Transkriptor, que utiliza Inteligencia Artificial (IA) para transcribir audios a textos^8^. Sin embargo, para garantizar la calidad de los datos, todo el material se revisó varias veces para corregir cualquier fallo. No se devolvieron transcripciones a los participantes.

De acuerdo con el concepto de Ciencia Abierta, todo el conjunto de datos de este artículo, es decir, las entrevistas completas, está disponible en el repositorio de datos de SciELO bajo el mismo nombre que este artículo, a través del enlace https://doi.org/10.48331/scielodata.0FSQFM.

El análisis de datos se realizó según el análisis temático de Braun y Clarke. Este proceso se llevó a cabo dentro de la plataforma Taguette, una herramienta gratuita que facilita la codificación, categorización, identificación de patrones y generación de informes^9^.

La familiarización con la base de datos se produjo durante la transcripción de las entrevistas, seguida de la lectura completa del material. Las fases dos y tres implicaron la creación de códigos y temas iniciales, centrándose en las individualidades de cada entrevista. En la cuarta fase, se realizó un nuevo análisis para refinar las categorías, considerando el conjunto de entrevistas. Se observó entonces la relación entre las categorías y las siete dimensiones de accesibilidad propuestas por Sassaki, lo que llevó a un nuevo análisis para verificar la adecuación de los hallazgos a estas dimensiones^10^.

### Consideraciones éticas

El estudio se llevó a cabo conforme a las directrices éticas nacionales e internacionales y fue aprobado por el Comité de Ética en Investigación del Hospital Israelita Albert Einstein, CAAE: 77221424.0.0000.0071, opinión n.º 6.885.669.

Los participantes fueron identificados mediante un código alfanumérico (por ejemplo, P1, P2...), omitiendo cualquier información sensible de las transcripciones.

## Resultados

Al final, se realizaron ocho entrevistas. Las edades de los participantes oscilaban entre 26 y 39 años, con una media de 31 años. Seis participantes son enfermeros y dos son técnicos de enfermería.

Los grados de sordera reportados variaron desde leves hasta profundos, con solo tres personas que adquirieron la condición antes de aprender a hablar. Solo un participante tenía Libras como lengua materna y no portugués. Esta misma persona era la única que se comunicaba únicamente a través de la Libras.

Dos participantes informaron conocer Libras, tres solo lo básico y tres no tener conocimientos de este idioma. Solo dos personas no usaron audífonos, las otras cinco los usaron en ambos oídos. Todos los entrevistados declararon que leen los labios, usando este recurso con poca o gran frecuencia, según sus necesidades.

En cuanto a la carrera de los participantes en enfermería, la duración de la experiencia osciló entre ocho meses y 17 años, con una media de 6,8 años. Cuatro trabajaron en el sector terciario de la salud y uno en el secundario. Para tres participantes, esta pregunta no se aplicaba, ya que uno trabajaba en un sector de salud gubernamental y dos eran profesores. Finalmente, los participantes procedían de las regiones Noreste, Sur y Sureste de Brasil.

En total, el análisis pasó por tres versiones hasta alcanzar un resultado satisfactorio. De las siete dimensiones de accesibilidad^6^, solo cinco eran aplicables a los resultados obtenidos en las entrevistas. Sin embargo, este artículo pretende tratar únicamente las dimensiones actitudinales y comunicativas. Estas dimensiones se dividieron entonces en accesibilidad y barreras. Es esencial enfatizar que los temas se construyeron a partir de los resultados obtenidos y no previamente establecidos. Además, las descripciones finales de estos se basaron en los conceptos presentados por Sassaki y se adaptaron según las necesidades del análisis.

Los temas son Accesibilidad y Barreras. El primero se refiere a los informes en los que fue posible identificar contextos alineados con los esfuerzos de inclusión. El segundo está relacionado con informes en los que fue posible identificar barreras que dificultaban la inclusión. 

Del primer tema derivan los subtemas Accesibilidades Actitudinales con 34 códigos y Accesibilidades de la Comunicación con 40 códigos. Del tema Barreras derivan los subtemas Barreras de actitud con 19 códigos y Barreras de comunicación con 24 códigos.

### Accesibilidades actitudinales

Demuestran actitudes y comportamientos favorables a la inclusión, como la representatividad, el apoyo y la convivencia en igualdad. Algunos ejemplos de código son:

*Al final, dije: “[...] Soy su enfermera, pero también tengo problemas de audición.” [...]. Los abuelos de ese niño entraron y dijeron: “La DA no va a impedir que mi nieto sea quien quiera ser.” (P6)*

*[...] Creo que cuando la persona lo quiere y hay otras personas que ayudan y se adaptan, siempre es posible. (P5)*

*A menudo, las chicas [el equipo de enfermería] me decían que solo después de tener contacto conmigo supieron cómo actuarían con estas pacientes [con DA], con las familias de esas pacientes. (P6)*

*[...] hoy en día todos mis pacientes, cuando digo, “Tengo discapacidad auditiva [...]”, todos lo aceptaron. (P6)*

*Creo que no tendría el nivel de empatía que tengo hoy si no hubiera tenido esta condición. (P1)*

### Accesibilidades de la comunicación

Demuestran el establecimiento de la comunicación entre el participante y otros profesionales, pacientes y sus familias, mediante la adopción de diferentes estrategias. Algunos ejemplos de código son:

*[...] Cuando llegué al sector, tuve que decirle a todos [...] “No oigo bien, si quieres hablar conmigo, háblame desde el frente o tócame, y entonces puedo hablar contigo, porque si me hablas desde atrás, no podré entenderlo.” (P1)*

*[...] Le dije: “Mira, ya que crees que mi AD está afectando así, entonces, a partir de ahora, te pido que me des por escrito todo el turno de todos los pacientes.” (P5)*

*Fui a ver a un paciente y le pregunté si podía quitarse la mascarilla, quedarse solo con la visera [pantalla facial transparente] y mantener la distancia. Tenía miedo, pero se lo quitó. (P2)*

*Cuando voy a comunicarme con personas mayores, uso más gestos, porque ya son mayores y luego no pueden leer por su visión. (P7)*

### Barreras actitudinales

Demuestran actitudes y comportamientos desfavorables a la inclusión, como capacitismo, invisibilidad de la sordera y discriminación. Algunos ejemplos de código son:

*Y también en el ámbito laboral, porque desafortunadamente existe un capacitismo en el que la gente piensa que, por ser una persona con discapacidad, eres incapaz o menos. [...] No tiene tanta habilidad como alguien que escucha. (P1)*

*Creo que debería haber más margen para recibirnos, y no menospreciar, porque aunque no lo parezca, sigo siendo una persona discapacitada. (P3)*

*Sabía [...] que había colegas que me preguntaron si podía ir a otro sector porque no podría estar en ese sector, ya que era una persona que no escucha. (P1)*

*Entonces tuve algunas dificultades, realmente, con estos temas [comprensión de otros profesionales sanitarios respecto a la DA] y pedí ayuda a la coordinación. ¿Tuvo buena gente? No necesariamente. (P3)*

*[...] Hubo una paciente que quiso medirle la tensión arterial [...] y dijo: “¿Vaya, solo hay una enfermera en la consulta externa y aun así puedes poner a una mujer sorda en mí?”. (P5)*

*Hacer activismo sobre eso es agotador, muy agotador (P2)*

### Barreras de comunicación

Presentan barreras de comunicación causadas o agravadas por la DA, como el uso de mascarillas protectoras, ambientes ruidosos, uso de la escritura y fatiga auditiva. Algunos ejemplos de código son:

*[...] Es un entorno [hospital] donde todos llevaban mascarilla, así que mi poder para leer los labios terminaba ahí. (P3)*

*El audífono nunca será un oído. ¡El dispositivo potencia enormemente todos los sonidos, todos ellos! Así que el ruido del aire acondicionado, para mí, será muy fuerte, pero el ruido de la voz, para mí, no será fuerte. (P3)*

*[...] Veo que todavía hay cierta barrera. Por ejemplo, trabajo en hemodinámica, y sabemos que [...] hay ruidos. Tiene una alarma de bomba, una alarma de monitor, una alarma de cardioversor [...]. (P6)*

*Además, sabes que las personas sordas a menudo escriben el portugués de un extranjero. No es el portugués convencional como escribe todo el mundo. (P7)*

*[...] Una persona que no oye bien y es profesional de la salud, lamentablemente, ya es mucho más ansiosa y cansada. [...] Porque hacemos un mayor esfuerzo que otras personas para entender la información [...]. (P4)*

*Mi mayor reto es trabajar en los sectores más confusos, como la UCI y urgencias, zonas con mucho movimiento, donde la gente no tiene tiempo de parar, muchas veces, para hablar conmigo, para prestar atención. (P7)*

## Discusión

La discusión de los temas se organizó por tamaño. Inicialmente, se presentarán las formas de accesibilidad observadas, seguidas de las respectivas barreras en cada una de ellas. Esta estructura permite comparar directamente los dos escenarios, facilitando una visualización más clara e integrada de cada dimensión analizada.

### Accesibilidades actitudinales

Un tema frecuente en los informes eran los escenarios de representación. También conocida como representación de identidad, consiste en personas que actúan como referencia para una población, ya que tienen características similares a las de los individuos representados^11^. La presencia de estas personas en las esferas sociales aumenta la conciencia social sobre las PCD y la inclusión, como se indica en la interacción de la P6 con la familia del niño.

A veces, los pacientes sufren condiciones que pueden experimentar los profesionales que les ofrecen atención. En otras palabras, la inclusión y la diversidad en la salud aportan avances sustanciales en la atención y experiencia del paciente^12^. También es importante destacar que la presencia de profesionales cuyo idioma y cultura son los mismos que los de sus pacientes colabora con la reducción de desigualdades en el acceso a la salud. Además, ayuda en este proceso promoviendo la educación y la concienciación de otros profesionales sobre PCD^13^.

Otro punto que se destacaba constantemente fue la importancia y el valor de recibir apoyo de otros profesionales y la coordinación de las instituciones. Este apoyo, especialmente cuando se realiza de manera genuina y no porque se considere incapaz o por lástima, fomenta una mayor autoconfianza y estabilidad en el profesional con discapacidad^14^. Sassaki afirma que una de las consecuencias de la inclusión es que este proceso acaba beneficiando no solo a la persona con discapacidad, sino también a la comunidad^5^ ^-^ ^6^, como ejemplifican los participantes 5 y 6.

En cuanto a los pacientes y su aceptación de recibir atención ofrecida por un profesional sanitario con DA, aunque hay informes de recepción negativa, la mayoría de los entrevistados reportaron escenarios positivos, como ejemplifica P6. En un estudio compuesto por profesionales de la salud sordos de diferentes áreas, se evidenció que, en general, los pacientes mostraron reacciones neutrales o positivas a la atención proporcionada por estos profesionales^12^.

Además, una característica relacionada con la atención ofrecida es la empatía del profesional con sus pacientes. Un estudio demostró que los estudiantes de medicina con diferentes discapacidades tenían menos probabilidades de tener bajos niveles de empatía^15^, una característica señalada por P1 como un punto positivo que la sordera aportó a su práctica de enfermería. Por lo tanto, los resultados de ambos estudios coinciden con los obtenidos en esta investigación.

### Barreras actitudinales

Cualquier persona puede enfrentarse a desafíos en el mercado laboral. Sin embargo, quienes tienen discapacidad sufren el *minority tax* que, en traducción libre, sería “tasa para minorías”. Esto significa que, además de las responsabilidades y dificultades naturales a este entorno, PCD afrontan obstáculos adicionales debido a prejuicios, aislamiento, exclusión, entre otros^12^.

Uno de los componentes de este fenómeno es el capacitismo. Este término se refiere a juzgar y discriminar a una persona en función de sus capacidades corporales y mentales, y a utilizar esto para clasificarla como incapaz^2^. Desafortunadamente, algunos entrevistados, así como P1, informaron haber experimentado este fenómeno. Es importante enfatizar que es una construcción estructural de la sociedad, resultado de un sistema histórico de políticas, instituciones y normas sociales, que devalúan y marginan a las PCD^2^. Los informes indican que puede provenir de una iniciativa institucional, como, por ejemplo, no proporcionar accesibilidad comunicativa, o puede ser a nivel individual, mediante actitudes negativas, como el uso de lenguaje discriminatorio.

Los estudios sobre la discriminación y el capacitismo que sufren los profesionales sanitarios con discapacidad demuestran que este escenario es recurrente. Además, puede desanimar a estas personas a trabajar en el sistema sanitario, o incluso afectar a su bienestar^2^. Afortunadamente, en el caso de los participantes en esta investigación, aunque generó cierta molestia, no hubo complicaciones importantes derivadas de actitudes capacitistas.

Otro factor relevante es la invisibilidad de la DA. No se puede distinguir a una persona con DA de otras personas a menos que decidan revelar esta información. A veces, el uso de implantes auditivos es una de las pocas formas de hacer visible esta característica^16^. Uno de los entrevistados (P3) informó que, debido a la invisibilidad de la sordera, otras personas tenían la percepción errónea de que la DA genera impactos mínimos en su vida profesional. 

Este informe muestra que la invisibilidad de la sordera puede generar formas sutiles de negación de la discapacidad, reforzando el capacitismo y creando obstáculos adicionales para la plena inclusión de personas sordas en la sociedad.

Otro obstáculo, un componente del *minority tax* y uno de los más importantes, es la discriminación. Se reportaron varios informes de discriminación sobre diversas interacciones con otros profesionales de la salud (P1), coordinación de instituciones (P3), pacientes y sus familias (P5).

Estos informes reflejan la falta de PCD en los espacios sociales y la ignorancia de la gente sobre estas personas. Existen varios estereotipos sobre las capacidades de una persona sorda, como ser menos inteligente, tener menos habilidades de comunicación, no tener habilidades sociales, no tener capacidad para escribir, entre otros^12^. Sassaki propone que uno de los principales medios para combatir la discriminación es la educación sobre la diversidad e inclusión de las PCD en todos los ámbitos de la sociedad. Una de las recomendaciones son los programas de concienciación dirigidos a los empleados, que han demostrado resultados positivos en la reducción de las barreras actitudinales en el entorno laboral^6^.

Por último, es esencial enfatizar que esta concienciación debe hacerse a partir de la iniciativa de las organizaciones, ya que a menudo solo corresponde a la persona con discapacidad realizar esta educación en su lugar, defendiendo su propio camino^6^ ^,^ ^12^. La necesidad de realizar autodefensa es uno de los componentes del *minority tax* y, al ser una demanda constante, se vuelve exhaustiva^12^, como menciona P2. En este sentido, conviene recordar que la verdadera inclusión requiere colaboración y esfuerzo mutuo entre las PCD y la sociedad^5^.

### Accesibilidades de comunicación

Un eje temático importante identificado fue la incorporación de estrategias de comunicación por parte de otras personas, según las necesidades específicas expresadas por cada participante, ejemplificado por P1. Es posible identificar que, en ciertas situaciones, acciones pequeñas y fáciles de implementar, como situarse delante al hablar con una persona sorda, pueden hacer que la comunicación sea accesible. Sin embargo, cabe señalar que se observó nuevamente que las PCD necesitan concienciar e instruir a otras personas sobre los métodos existentes para generar inclusión, ya que muchas no disponen de este conocimiento^5^ ^,^ ^12^. Por tanto, reforzar la asimetría en el esfuerzo realizado por la inclusión, como describe Sassaki en sus análisis de prácticas integrativas^5^ ^-^ ^6^.

Una característica específica de la rutina de los profesionales de enfermería es el cambio de turno, que normalmente se realiza verbalmente. Sin embargo, se hizo un ajuste en la forma en que se transmitía la información después de que el entrevistado P5 informara de tener dificultades con este proceso, ya que el escenario consistía en varias personas hablando al mismo tiempo.

Este ejemplo reafirma lo presentado en un estudio con personas con pérdida auditiva unilateral, que destacó que los entornos ruidosos afectan la capacidad del individuo para entender el habla^17^ ^-^ ^18^. Además, en un escenario como el descrito por el participante, con varias personas hablando al mismo tiempo, no es posible leer los labios a todos. Así, cambios como el mencionado pueden aportar mayor seguridad tanto al profesional como al paciente, ya que la comunicación eficaz es crucial para una atención adecuada^19^.

Finalmente, los entrevistados informaron de algunas estrategias que desarrollaron para establecer una comunicación efectiva con sus pacientes, como, por ejemplo, la lectura labial y los gestos en el caso de P2 y P7, respectivamente. Los estudios presentan otras estrategias utilizadas por profesionales de la salud con sordera para establecer comunicación con sus pacientes. Algunos ejemplos son: sistemas de micrófonos, máscaras inclusivas, aunque no estandarizadas, intérpretes, tarjetas explicativas y aplicaciones de transcripción simultánea de texto^19^.

Se puede inferir que la elección de la estrategia utilizada depende de múltiples factores, como la disponibilidad de recursos, las necesidades del profesional sordo y las especificidades de cada paciente. Por tanto, no hay forma de establecer un método ideal estandarizado. Por ello, deben existir varias formas de comunicación disponibles, para que el profesional pueda elegir las que mejor se adapten a cada caso. 

### Barreras de comunicación

En varios entornos de trabajo de enfermería se utilizan la mayoría de las veces mascarillas protectoras. Durante la pandemia de la COVID-19, este uso se volvió aún más necesario y ocurrió en situaciones en las que, a diario, no era habitual, generando impactos significativos para PCD auditiva^20^.

Como ejemplifica P6, el uso de mascarillas afecta a la capacidad del individuo para leer los labios, lo que suele complementar la comunicación. En el caso de las personas sordas que usan Libras, el uso de mascarillas impide la percepción de las expresiones faciales, que son importantes en la lengua de signos^20^. Por ello, el uso de mascarillas generó mayores niveles de estrés y ansiedad en los profesionales de la salud con DA. Algunos participantes señalaron que esto les hacía temer el futuro de sus carreras, lo que les llevaba a cuestionarse si tendrían que cambiar de profesión, ya que esta barrera parecía imposible de superar. Por lo tanto, esta barrera también afectó el sentido de autonomía, independencia y pertenencia de estos profesionales, favoreciendo una sensación de aislamiento e incapacidad para demostrar competencia profesional^20^.

Otro factor que dificulta la comunicación es la percepción auditiva en entornos ruidosos. Algunos ejemplos donde esto ocurre, según los participantes, serían centros quirúrgicos, urgencias, UCIs, hemodinámicas, entre otros. Con frecuencia, las personas que usan audífonos informan tener dificultades con la discriminación del habla auditiva en entornos acústicamente desafiantes. En estos casos, corresponde al individuo dedicar un mayor esfuerzo cognitivo para distinguir el habla de otros ruidos. Este esfuerzo requiere una alta carga de concentración, que ya no está destinada a otras actividades^21^.

En cuanto al uso de la escritura como estrategia de comunicación, es importante enfatizar que, aunque puede ayudar en algunas situaciones, este método puede no ser completamente eficaz. Aquellas personas con sordera que han escrito portugués como segunda lengua pueden presentar dificultades con este modo de comunicación, debido a las diferencias semánticas y gramaticales entre Libras y portugués^19^. En el caso de personas con DA que tienen el portugués como lengua materna, se considera que pueden presentar dificultades para aprender el idioma, hablado y escrito, como resultado de la DA^17^ ^-^ ^18^. En vista de esto, la comunicación escrita no debe considerarse como el principal medio para establecer una comunicación efectiva con una persona con sordera^19^.

Teniendo en cuenta todas estas barreras de comunicación, participantes como P4 reportaron sentir fatiga mental como resultado del esfuerzo continuo por poder comunicarse. La fatiga auditiva también es reportada por otras personas con sordera tras estar en ambientes ruidosos, que exigen interactividad y, por tanto, una mayor carga cognitiva y de concentración. Como resultado, puede provocar niveles más altos de estrés ^17^ ^-^ ^18^. Sin embargo, esta fatiga mental no fue la única consecuencia identificada en las barreras de comunicación. Los participantes informaron como P7 que ciertas áreas de la actividad de enfermería empezaron a considerarse inviables para profesionales con DA, debido a las barreras encontradas, especialmente las de comunicación.

Este informe ejemplifica la fase de integración, ya que las personas con DA entran en entornos sin recibir las adaptaciones necesarias. Por tanto, estas personas deben adaptarse en la medida de lo posible^4^. Es común que, aunque sea de forma involuntaria, las instituciones acaben desanimando a estas personas a trabajar en subespecialidades. Esto se debe tanto al hecho de que no proporcionan las adaptaciones necesarias, como a la creencia de que estas personas no deberían o no podrían estar en espacios de contacto con el paciente, o donde la comunicación debería ser rápida. Sin embargo, con los recursos necesarios, es posible que las personas con DA ejerzan su profesión con calidad y seguridad^12^.

Este estudio tenía como limitación un número de participantes menor de lo esperado, ya que se estimó en 15 individuos. Sin embargo, esta dificultad ya estaba prevista dadas las limitaciones de acceso a esta población^22^. En cualquier caso, se cree que fue posible obtener resultados significativos sobre el tema y ofrecer una visión innovadora del desempeño de los profesionales de enfermería sordos, ya que cualquier propuesta sobre las PCD debe involucrar a las mismas.

## Conclusión

Al comparar el concepto de inclusión presentado por Sassaki con las experiencias de los participantes, es posible entender que, en las dimensiones de la accesibilidad actitudinal y la accesibilidad comunicacional, la inclusión plena de las personas con DA en enfermería aún está lejos de lograrse. La ignorancia de la sociedad sobre las PCD y los medios existentes para mitigar las barreras es una de las principales causas de los desafíos identificados. 

Sin embargo, los informes positivos demuestran que esta es una realidad en construcción. Por ello, cabe mencionar que es imprescindible que la sociedad tenga un papel más activo en el proceso de inclusión de las PCD, para que no recaiga solo en ellas.

Por otro lado, la presencia de PCD auditiva en enfermería contribuye a la conciencia social sobre las PCD, demostrando que la discapacidad no impide la práctica de la enfermería, rompiendo estigmas y prejuicios y reforzando la importancia de implementar estrategias de accesibilidad. En consecuencia, esta presencia ayuda a reducir las desigualdades en el acceso a la salud para otras PCD. En la dimensión comunicacional, es posible entender que, con las adaptaciones necesarias, la comunicación se vuelve factible para que los profesionales desempeñen sus funciones con seguridad, autonomía y niveles más bajos de esfuerzo mental.

## Data Availability

El conjunto de datos de este artículo está disponible en la página de la RLAE en el repositorio SciELO Data, en el enlace https://doi.org/10.48331/scielodata.0FSQFM.
